# The synergistic impact of Spirulina and Sulfate reducing bacteria on lettuce growth in Cadmium contaminated soil

**DOI:** 10.1038/s41598-025-85996-y

**Published:** 2025-02-21

**Authors:** Maryam Seifikalhor, Mojgan Latifi, Neda Nasiri Almanghadim, Zahra Akbar-Tajari, Anahita Ahangir, Seyedeh Batool Hassani, Neda Soltani, Hossein Sadeghi, Elyas Eghbal, Zahra Fallahi, Nazim S. Gruda

**Affiliations:** 1 Department of Molecular Physiology, Agricultural Biotechnology Research Institute of Iran (ABRII), Agricultural Research, Education and Extension Organization (AREEO), Karaj, Iran; 2 Department of Plant Sciences and Biotechnology, Faculty of Life Sciences and Biotechnology, Shahid Beheshti University, Tehran, Iran; 3 Department of Petroleum Microbiology, Research Institute of Applied Science, Academic Center for Education, Culture and Research (ACECR), Tehran, Iran; 4 Department of Horticulture, College of Aburaihan, University of Tehran, Tehran, Iran; 5 Department of Microbiology and Microbial Biotechnology, Faculty of Life Sciences and Biotechnology, Shahid beheshti University, Tehran, Iran; 6 Institute of Plant Sciences and Resource Conservation, Division of Horticultural Sciences, University of Bonn, 53113 Bonn, Germany

**Keywords:** Agricultural productivity, Contamination, Detrimental effects, Heavy metal, Photosynthetic capacity, Plant growth, Plant sciences, Environmental sciences

## Abstract

Cadmium (Cd) contamination is a critical environmental issue, adversely affecting plant growth and agricultural productivity. While numerous studies have explored the role of various bacteria in mitigating heavy metal toxicity, the specific impacts of sulfate-reducing bacteria ( *Desulfovibrio desulfuricans*, *SRB*) and the cyanobacterium Spirulina (*Arthrospira platensis*, *SP*), both individually and in combination, on Cd-contaminated plants remain underexplored. This study investigates the effects of SRB and SP on lettuce plants exposed to Cd contamination, aiming to enhance our understanding of their potential in alleviating Cd toxicity and promoting plant health. Results revealed that Cd contamination significantly reduced root growth in all treatments except for the combined application of SRB and SP. This combination also led to a marked decrease in leaf Cd content and improved leaf area, particularly under Cd stress. Furthermore, SP and SRB together increased the relative water content in contaminated soils, and SRB alone induced hydrogen peroxide production in non-contaminated soils. The co-application of SRB and SP significantly boosted catalase and superoxide dismutase activities, enhancing photosynthetic capacity and overall plant growth under Cd stress. These findings underscore the promising potential of using SRB and SP synergistically to mitigate Cd-induced challenges in lettuce cultivation, offering a viable strategy to improve crop productivity in contaminated environments.

## Introduction

Cadmium (Cd) is a highly toxic heavy metal that presents a grave threat to ecosystems and human health, owing to its pervasive presence and long-lasting persistence in the environment. The advent of industrialization has significantly increased agricultural productivity; however, it has also resulted in the extensive degradation of the environment^1–3^. The indiscriminate use of chemical fertilizers to improve plant health, productivity, and disease control has disrupted the delicate ecological balance of the soil and led to the depletion of essential nutrients and exchange with harmful elements^4,5^. The overuse of chemical fertilizers to improve plant growth and productivity has disrupted the delicate balance of soil ecosystems. This imbalance depletes essential nutrients and facilitates their replacement with harmful elements such as Cd. As a result, Cd is released into the environment, where it accumulates in soils and water bodies, eventually entering the food chain and posing serious ecological and health risks. The detrimental effects of Cd on various organisms, including microorganisms, plants, animals, and humans, have been extensively studied^6^.

Iran has experienced significant industrial growth recently, including mining, metal smelting, and manufacturing. These industries often involve Cd-containing materials or processes that release Cd into the environment as a byproduct. Moreover, Iran is known to have geological formations and natural deposits that contain elevated levels of Cd. Weathering and erosion of these geological formations can release Cd into the surrounding environment, contributing to Cd pollution in certain regions. Recent studies have highlighted urban dust from traffic and building erosion, potentially contributing to heavy metals such as Cd contamination in underground water during the rainy season. This emerging issue has gained attention recently due to its implications for widespread heavy metal pollution^7,8^. Given the increasing pollution caused by Cd, exploring potential solutions for utilizing polluted agricultural fields is crucial. Recently, various biofertilizers have emerged as a promising solution due to their environmentally friendly, non-toxic, and sustainable nature^8–11^. They are products composed of carefully selected and beneficial living microorganisms used as microbial inoculants for soil application. A wide range of microorganisms, including Cyanobacteria, Azolla, Bacillus, Rhizobium, endophytic diazotrophs, and phosphate-solubilizing microorganisms, are being extensively investigated and employed as biofertilizers^12^. These microorganisms can improve plant health through the secretion of growth-promoting substances.

While certain plants, such as the hyperaccumulator *Solanum nigrum*, have been shown to accumulate significant amounts of cadmium, its detrimental effects on the growth of most plant species are well-documented^13^.Using microorganisms has emerged as a promising strategy for mitigating Cd contamination^14–16^. Spirulina (SP) is a filamentous cyanobacterium known for its exceptional protein content and nutritional value. SP can thrive in diverse environments, including Cd-contaminated sites^17–19^. Its unique ability to tolerate and accumulate heavy metals makes it a potential candidate for phytoremediation applications^20^. Moreover, sulfate-reducing bacteria (SRB) have also shown promise in heavy metal detoxification^19,21,22^. SRBs are anaerobic bacteria that play a crucial role in the environment. These bacteria can utilize sulfate as an electron acceptor during respiration, effectively reducing it to sulfide. This metabolic process contributes to sulfur’s biogeochemical cycling and has significant implications for various natural systems, including metal sedimentation^23^. This function may result in the precipitation of Cd as insoluble Cd sulfide (CdS), reducing the solubility, bioavailability, and toxicity of Cd. The toxicity of Cd is primarily associated with its soluble form, as it is more bioavailable and can be readily absorbed by organisms. However, by converting soluble Cd into insoluble forms, SRB significantly decreases its bioavailability and, consequently, its toxicity. The interaction between SP and SRB in reducing Cd contamination has gained attention in current research due to their complementary mechanisms. SP sequesters Cd through intracellular accumulation and extracellular binding, while SRB contributes to the immobilization of Cd as insoluble CdS, further limiting its mobility and potential harmful effects^24,25^. One of the well-known strategies employed for the remediation of Cd in plants involves using sulfur-rich components. These compounds are critical in empowering plants to fortify their cellular defenses and effectively sequester Cd within vacuoles, a process facilitated by the remarkable phytochelatins^26^. Sulfur, an indispensable macronutrient crucial for the optimal growth and advancement of plants, assumes a vital function in numerous cellular mechanisms. It actively participates in diverse processes, including protein biosynthesis, chlorophyll formation, and defense mechanism^26,27^. Sulfids produced by the SRB metabolic activity react with the metal to form water-insoluble metal-sulfides, making it insoluble in water^2,28^. The presence of SRB in soil contaminated with Cd will facilitate the reduction of sulfate to sulfide, resulting in increased hydrogen sulfide production (H_2_S). We hypothesize that the interaction between H_2_S and Cd will lead to the formation of insoluble Cd compounds, reducing the bioavailability and mobility of Cd in the soil. Additionally, we propose that the co-application of SP, known for its metal sequestration and plant growth-promoting abilities, will further enhance the remediation of Cd-contaminated soil and promote the growth of Cd-tolerant plants through synergistic effects with SRB-mediated sulfide production. Due to the limited availability of evidence regarding the influence of SRBs on plants and their potential as a pathogen, SP can be considered a pathogen suppressant, effectively preventing the inhibition of plant growth.

In this study, we assess SRB effect on lettuce (*Lactuca sativa*) growth and physiological responses in the presence of Cd, both with and without SP as it is a widely studied plant species that could serve as a model organism for investigating various aspects of plant biology, including genetics, physiology, development, and responses to environmental cues^29^. Lettuce also exhibits a diverse range of morphological traits, which allows researchers to explore developmental processes such as leaf formation and root architecture^30–34^. Additionally, lettuce’s responses to biotic and abiotic stresses, such as pathogens, drought, and salinity, have been extensively investigated, providing insights into plant defense mechanisms and stress tolerance^35^.

The findings of this study could contribute to the development of innovative biotechnological approaches for Cd phytoremediation. By exploring the potential of these microorganisms, we aim to provide sustainable and efficient solutions for mitigating Cd contamination and reducing its detrimental effects on the environment and human health.

## Result

### Combined SRB and SP application showed a positive impact on root growth in contaminated soil

**Fig. 1:**
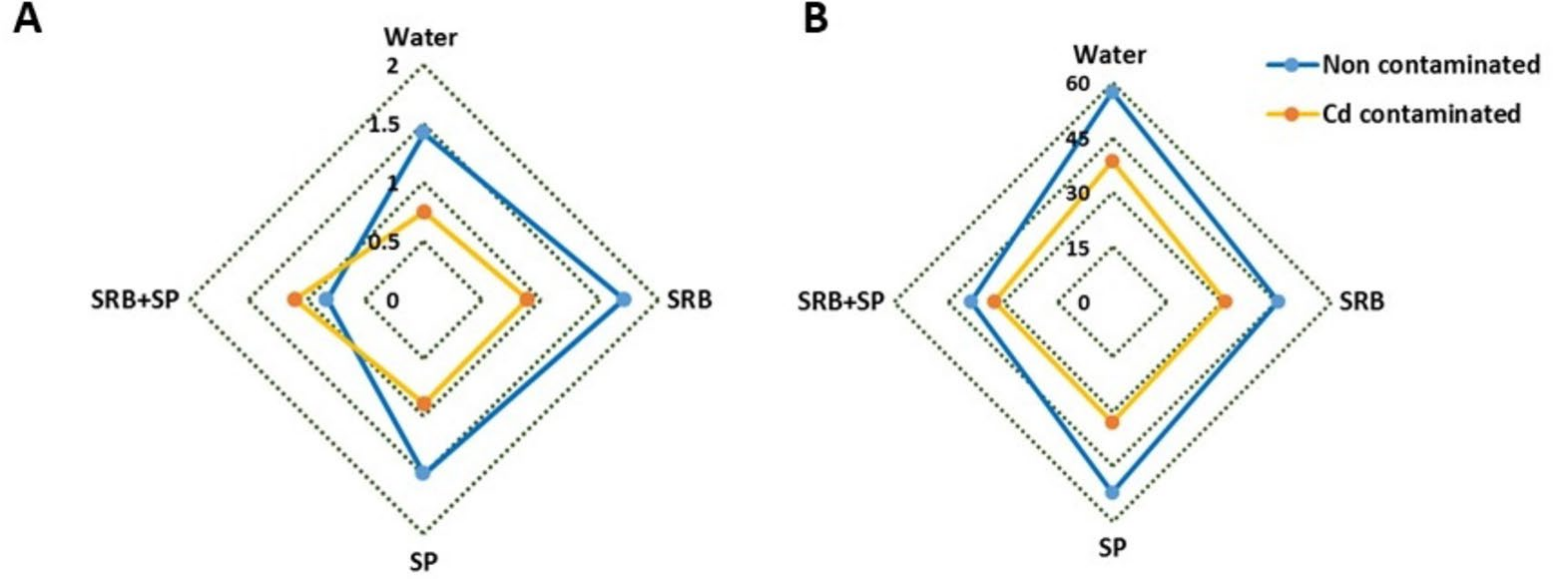
Spider plot of the Cd [0 mg/kg (control (blue line)) and 150 mg/kg CdNO_3_ (yellow line)] effect on lettuce root growth (**A**) and seed germination (**B**) eight weeks after sowing and treatment with water, sulfate-reducing bacteria (SRB), Spirulina (SP) and co-application of SRB and SP (SRB + SP). The values of the calculated parameters were shown as relative to those of the control plants (Water treatment).

Using SRB increased root growth while applying the SP, which did not yield a significant effect in non-contaminated conditions. However, when SP was combined with SRB, it led to a decline in root growth compared to the water-treated plants. The contamination with Cd caused a reduction in root growth compared to the control plants. However, no significant differences were observed among the treatments regarding their influence on root growth except for SRB and SP co-application, which increased root growth (Fig. 1A). Under noncontaminated conditions, only the combined application of Spirulina (SP) and sulfate-reducing bacteria (SRB) demonstrated adverse effects on seed germination. Conversely, in contaminated soil, all treatments exhibited a reduced germination rate (Fig. 1B).

### The co-application of SP and SRB demonstrated a beneficial effect on leaf area and relative water content in contaminated soil

**Fig. 2:**
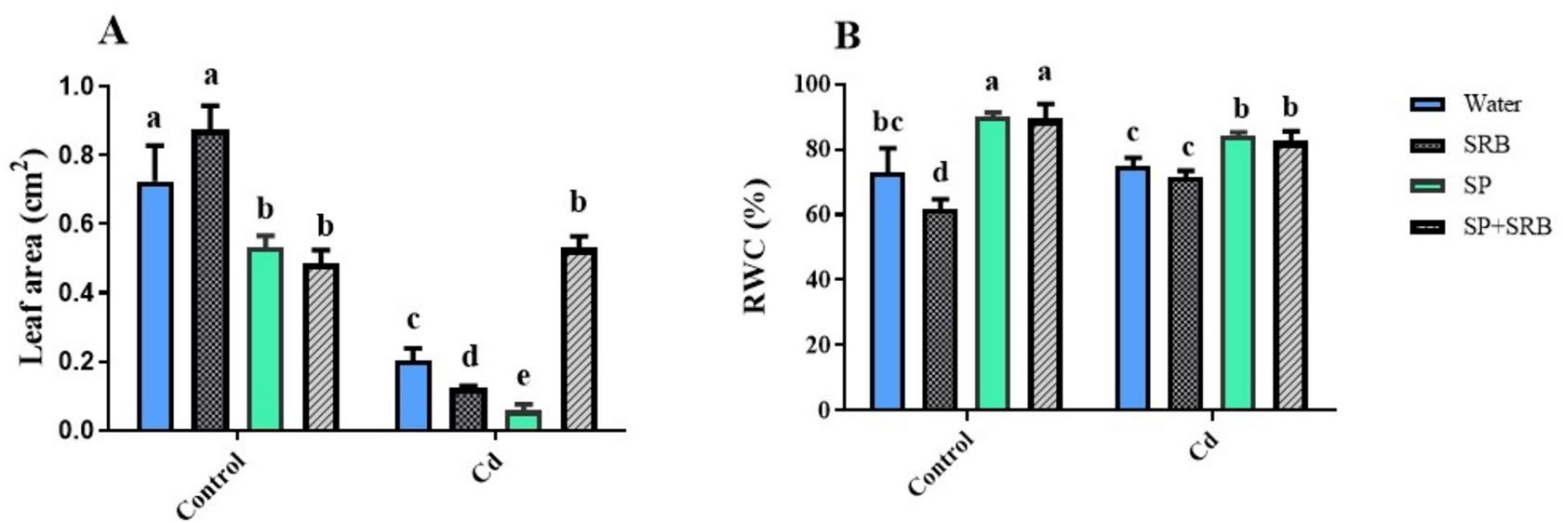
Effects of Cd [0 mg/kg (control) and 150 mg/kg CdNO_3_] on leaf area (**A**) and relative water content (RWC) (**B**) of lettuce plants over eight weeks after sowing and treatment with water, sulfate-reducing bacteria (SRB), Spirulina (SP) and co-application of SRB and SP (SRB + SP). Each column represents the mean value of three replications ± standard error. Different letters indicate a significant difference between the means at the probability level of *P* < 0.05.

In non-contaminated soil, the presence of SP alone or when combined with SRB resulted in a significant decrease in leaf area. In contrast, the application of the SRB positively affects leaf area. Under Cd contamination, the presence of SRB or SP alone reduced leaf area, whereas the combined application of SP and SRB resulted in a significant increase. However, overall, Cd treatment caused a notable decrease in leaf area compared to the control condition (Fig. 2A); the effect of SP and SRB co-application was similar to the noncontaminated condition.

Both SP alone and in combination with SRB significantly increased the relative water content (RWC) in non-contaminated conditions. Notably, in Cd-contaminated conditions, the simoltenus application of SP and SRB increased RWC compared to water-treated plants (Fig. 2B).

### Antioxidant enzyme activity enhanced by the combined application of SP and SRB

Under contaminated and non-contaminated conditions, the level of H_2_O_2_ showed no significant change, except when subjected to SRB treatment, which resulted in a substantial increase in its concentration in both conditions (Table 1). The activity of the SOD enzyme only increased in plants grown in non-contaminated soil when treated with SP.

In non-contaminated soil, the activity of the SOD enzyme increased only in plants treated with SP. In Cd-contaminated soil, however, SOD activity was significantly reduced in plants treated with water or SRB. Despite this, plants treated with SP alone or in combination with SRB exhibited an increase in SOD activity, suggesting a protective effect of SP against in both contaminated and non-contaminated conditions.

(Table 1). The CAT enzyme activity was significantly reduced under the noncontaminated soils in treatments with SRB. In contrast, SP treatment had a pronounced effect on its activity. However, despite an overall decrease in its activity under Cd contamination, SP alone or in combination with SRB showed an increase compared to water-treated plants (Table 1).

**Table 1:** Effects of cadmium (cd) [0 mg/kg (control) and 150 mg/kg CdNO_3_] on H_2_O_2_ content, superoxide dismutase (SOD), and catalase (CAT) activity of lettuce plants over eight weeks after sowing and treatment with water, sulfate-reducing bacteria (SRB), Spirulina (SP) and co-application of SRB and SP (SRB + SP).

Condition		H_2_O_2_	CAT	SOD
Non-contaminated	Water	0.225 ± 0.03 cd	0.033 ± 0.002 b	7.1 ± 0.13 cd
	SRB	0.722 ± 0.05 a	0.002 ± 0.0005 e	7.4 ± 0.13 c
	SP	0.27 ± 0.04 c	0.065 ± 0.006 a	8.45 ± 0.24 a
	SP + SRB	0.183 ± 0.03 d	0.008 ± 0.002 d	7.72 ± 0.07 c
Contaminated	Water	0.29 ± 0.01 c	0.01 ± 001 d	6.62 ± 0.15 f
	SRB	0.524 ± 0.02 b	0.001 ± 0.0005 f	6.54 ± 0.08 f
	SP	0.27 ± 0.04 c	0.013 ± 0.001c	7.49 ± 0.07 c
	SP + SRB	0.304 ± 0.03 c	0.043 ± 0.004 b	7.99 ± 0.15 b
P value Significant level	Biological treatment			*P* = 0.0064 *
	Cd contamination			*P* = 0.7703 ^ns^
	Biological treatment × Cd contamination			*P* < 0.0001****

*significant at P < 0.05; **** significant at P < 0.000; ns not significant. Different letters mean differ significantly (2-way ANOVA, P < 0.05).

### The simultaneous treatment by SP and SRB improved photosynthetic performance

**Fig. 3:**
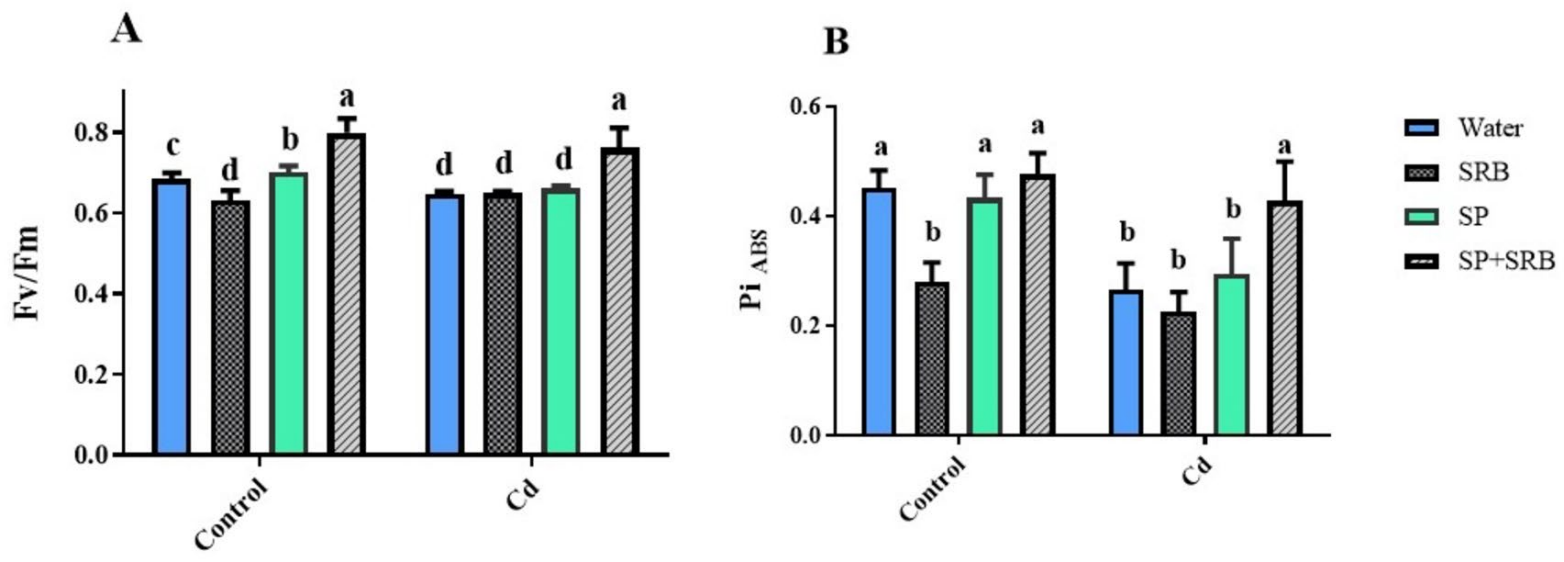
Effects of Cd [0 mg/kg (control) and 150 mg/kg CdNO_3_] on maximum quantum efficiency of photosystem II (F_v/_F_m_) (**A**) and performance index on absorption basis (Pi-_ABS_) (**B**) of lettuce plants over 8 weeks after sowing and treatment with water, sulfate reducing bacteria (SRB), Spirulina (SP) and co-application of SRB and SP (SRB + SP). Each column represents the mean value of three replications ± standard error. Different letters indicate a significant difference between the means at the probability level of *P* < 0.05.

In plants not exposed to Cd, the combined treatment of SP and SRB resulted in the highest maximum quantum efficiency of photosystem II (F_v_/F_m_) values, indicating the most favorable photosynthetic performance. Importantly, all treatments were ineffective in mitigating Cd’s negative impact on F_v_/F_m_ except for the SP and SRB combination that exhibited maximum F_v_/F_m_, implying a positive role in mitigating Cd contamination on photosynthesis performance (Fig. 3A).

In non-contaminated plants, the application of SRB decreased the performance index (Pi-abs), a parameter reflecting overall photosynthetic efficiency. Conversely, when SRB was applied with SP, a dramatic increase in Pi-abs was observed under Cd contamination conditions (Fig. 3B).

**Fig. 4:**
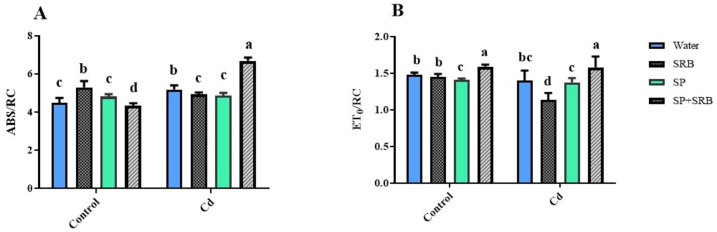
Effects of Cd [0 mg/kg (control) and 150 mg/kg CdNO_3_] on specific energy fluxes per reaction center (RC) for energy absorption (ABS/RC) (**A**) and trapped energy flux (TR0/RC) (**B**) of lettuce plants over 8 weeks after sowing and treatment with water, sulfate reducing bacteria (SRB), Spirulina (SP) and co-application of SRB and SP (SRB + SP). Each column represents the mean value of three replications ± standard error. Different letters indicate a significant difference between the means at the probability level of *P* < 0.05.

The ABS/RC value exhibited an increase in non contaminated conditions when treated with SRB. However, under Cd contaminated soil, the opposite trend was observed, and the simultaneous application of SP and SRB resulted in a significant increase in the ABS/RC value (Fig. 4A). Regarding the ET0/RC parameter, only the combination of SRB and SP exerted a positive influence, leading to a significant increase. However, the remaining treatments decreased ET0/RC under both contaminated and non-contaminated conditions (Fig. 4B).

### Cd content dramatically reduced in plants subjected to simultinous SP and SRB

**Fig. 5:**
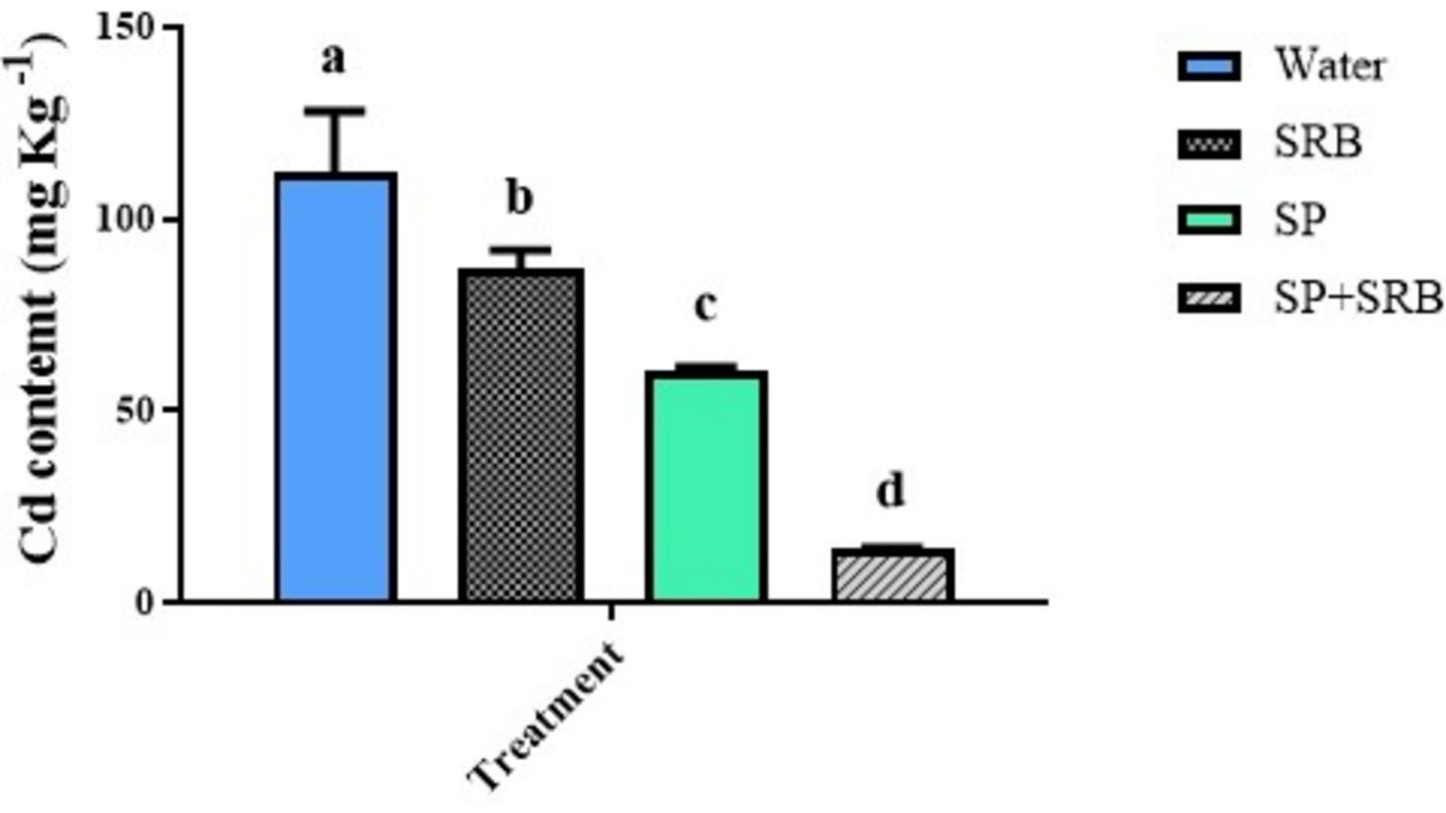
Effects of Cd [0 mg/kg (control) and 150 mg/kg CdNO_3_] on Cd content of lettuce plants leaves over eight weeks after sowing and treatment with water, sulfate-reducing bacteria (SRB), Spirulina (SP) and co-application of SRB and SP (SRB + SP). Each column represents the mean value of three replications ± standard error. Different letters indicate a significant difference between the means at the probability level of *P* < 0.05.

The Cd content measured in lettuce leaves showed the minimum content in plants grown under SP and SRB co-application. The maximum content was observed in plants subjected to water. SP also reduced the Cd content in plants when compared to the water-treated plants (Fig. 5).

## Discussion

We investigated the effects of different bacterial treatments, including SRB and SP, on lettuce plants exposed to Cd contamination. Cd exposure can disrupt various physiological processes, including nutrient uptake, photosynthesis, and cellular metabolism^36,37^. SRB activity might mitigate Cd contamination by detoxifying Cd or reducing its availability to plants, thereby improving plant growth and fresh weight in Cd-contaminated conditions. SRBs promote nutrient availability, particularly sulfur^38,39^. Sulfur is a vital nutrient for plants, serving an indispensable role in protein synthesis, enzyme activities, and overall plant growth. By enhancing sulfur availability, SRB treatment can improve nutrient uptake and utilization, increasing biomass production^40^. SP, known as a beneficial microorganism, stimulates the production of plant growth-promoting substances such as auxins, cytokinins, and gibberellins. These hormones play crucial roles in cell division, elongation, and differentiation, ultimately increasing fresh weight and biomass. Moreover, the application of SRB has shown promising potential in mitigating the negative impacts of Cd stress on plant growth^41–43^.

### Benefits of SP and SRB on plant development, root growth, leaf area, and water content

**Fig. 6:**
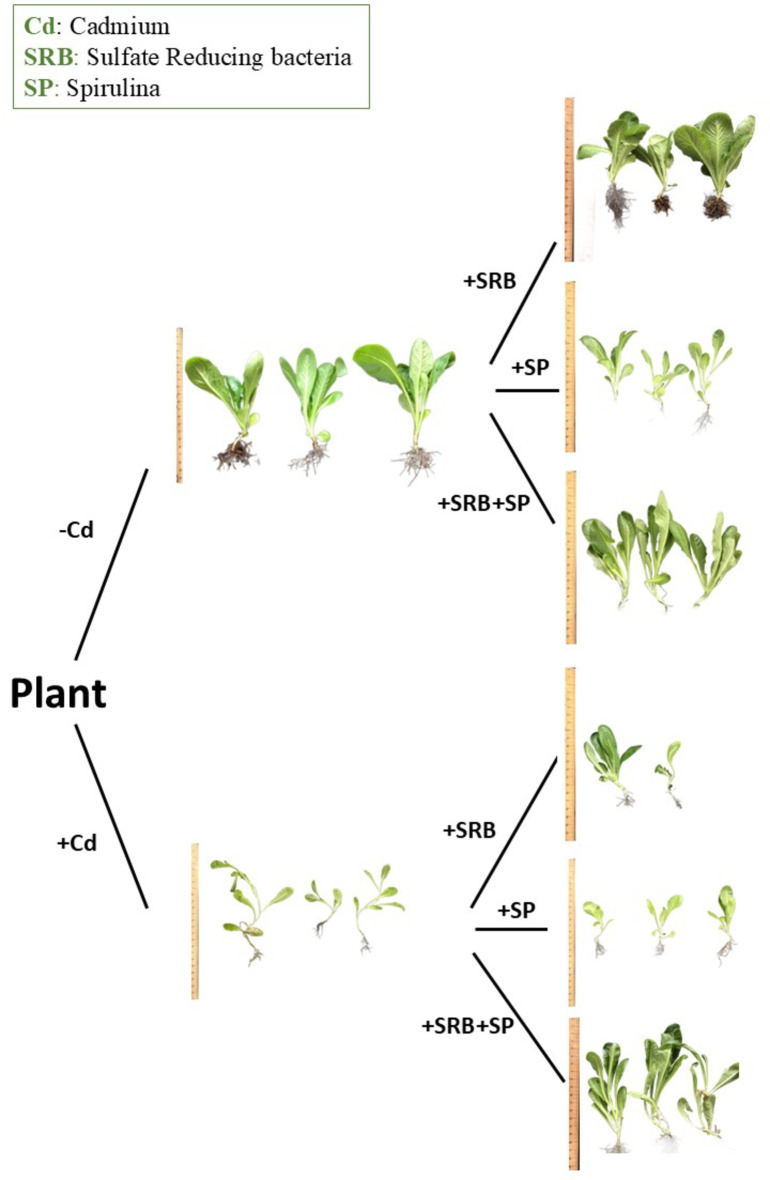
Schematic diagram showing the effects of *Spirulina* (SP), sulfate-reducing bacteria (SRB), and their combination on overall plant growth under Cd stress conditions. The figure depicts a representative plant under each treatment and Cd stress, highlighting differences in overall growth, leaf area. The diagram illustrates that SRB combined with SP significantly enhances plant growth under cd stress condition.

Root length is an essential indicator of plant growth and nutrient uptake capacity. In the absence of Cd stress, the application of SRB led to a significant increase in root length and overal plant growth (Fig. 6), suggesting its positive influence on root development. However, when SRB was combined with SP, root length was reduced compared to the control’s. Different scenarios can bring observed outcomes: (i) the combination of SRB and SP may result in interactions between microorganisms and plants that affect root development. These interactions could involve competition for resources or signaling molecules that regulate root growth. (ii) Adding SP and SRB may change the soil’s nutrient availability or alter the plant’s nutrient uptake mechanisms. This could impact root development and result in a reduction in root length. (iii) SRB and SP may modulate plant hormone signaling pathways differently when combined compared to when applied individually. Hormones, such as auxins and cytokinins, are essential in root growth and development. Changes in hormonal signaling could affect root elongation and reduce root length; (iv) The combination of SRB and SP might induce a stress response in the plant, inhibiting root growth. The plant may allocate resources towards stress response mechanisms, diverting energy from root development.

In our study, treatments involving SRB, or its combination with SP, demonstrate a negative influence on seed germination in both contaminated and non-contaminated conditions. However, an adverse effect was not observed with SP alone. This suggests that these treatments may not directly affect the germination process. However, it is noteworthy that the presence of Cd reduced germination percentage across all treatments. The effect of SRB on seed germination appears to differ from that of other plant growth-promoting bacteria (PGPB). While many PGPBs have been shown to positively influence seed germination by enhancing seedling establishment and vigor, the impact of SRB on seed germination is inconsistent with these findings^44^. Previous research has demonstrated that certain PGPB can promote seed germination through various mechanisms, including the production of phytohormones, nutrient solubilization, and protection against pathogens^45,46^. The impact of SRB on seed germination does not adhere to this observed trend. SRB may not exhibit the same influence on seed germination or may even have variable effects. This inconsistency could be attributed to several factors, such as differences in bacterial species, strains, or the specific plant species under investigation. Additionally, the mechanisms by which SRB interacts with plants may differ from those employed by other PGPBs, leading to distinct effects on seed germination.

Leaf area results show an opposite trend; low leaf area is observed with SRB treatment, and high leaf area is monitored with SP combination with SRB. According to their negative co-effect on root growth, trade-offs in resource allocation could be one of the critical possibilities. The positive effects of the SRB and SP combination in Cd contamination soil on leaf area or RWC may come at the expense of reduced allocation of resources towards root growth^47^. Occasionally, plants prioritize above-ground growth and reproductive processes over root development, reducing root growth despite overall positive effects on plant performance^48^. Although the microbial treatments, whether applied alone or in combination, may have shown worse results compared to the control group in due to several possible reasons. In the absence of cadmium (Cd) stress, the application of sulfate-reducing bacteria (SRB) and *Spirulina* may negatively impact plant performance due to several factors. These microbes can trigger unnecessary stress responses, diverting plant resources away from growth and development toward unneeded defense mechanisms. Additionally, their introduction can disrupt nutrient balance in the rhizosphere, as SRB’s production of sulfide and *Spirulina*’s alteration of soil chemistry may negatively affect nutrient availability. Competition between the introduced microbes and native soil microorganisms can further reduce nutrient access, while oxygen imbalances caused by *Spirulina*’s oxygen production and SRB’s anaerobic preferences may create unfavorable root-zone conditions. These combined effects can lead to worse outcomes compared to untreated controls.

### Modulation of catalase and superoxide dismutase cctivities by SRB and SP: implications for H2O2 regulation and plant development in cd contaminated soil

**Fig. 7:**
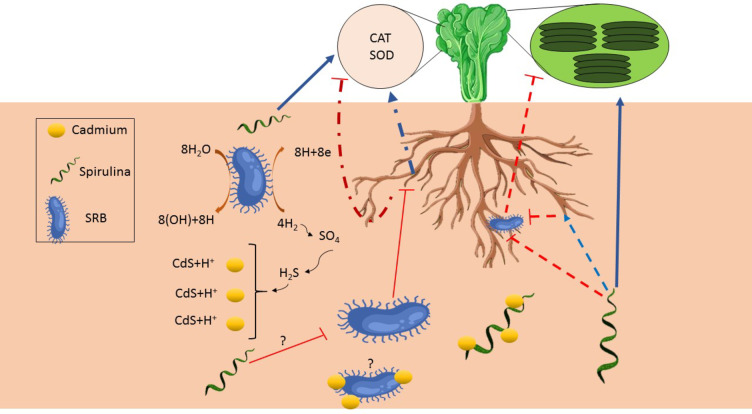
A schematic representation of the influence of Spirulina (SP) and sulfate-reducing bacteria (SRB) in the presence of cadmium (Cd) on lettuce root. The red lines indicate inhibitory effects. The blue lines represent positive effects, the dashed lines indicate hypothetical (positive or negative) effects, and the question marks indicate hypothetical unknown mechanisms. Green circle in the top right corner represents the chloroplast and refres to the photosynthetic activity.

H_2_O_2_content is an essential indicator of plant oxidative stress levels^49^. In our study, the application of SRB alone led to a significant increase in H_2_O_2_ content in both control and Cd conditions. This suggests that SRB treatment may induce oxidative stress in lettuce plants. In contrast, the other treatments, including SRB combined with SP, did not significantly affect H_2_O_2_ levels. Elevated H_2_O_2_can exert positive and negative effects on plant physiology, depending on the concentration and duration of exposure^50^. At low concentrations, H_2_O_2_is a signaling molecule that initiates various physiological responses in plants. It activates defense mechanisms against pathogens, triggers antioxidant systems, and regulates growth and development^51^. In this context, the increase in H_2_O_2_ induced by SRB could be a part of a signaling cascade that initiates plant responses as pathogen attacks. However, if the levels of H_2_O_2_become excessively high, it can lead to oxidative stress^49^. Oxidative stress arises from an imbalance between the excessive production of reactive oxygen species (ROS) and the body’s ability to counteract their harmful effects, including H_2_O_2_, and the plant’s ability to detoxify or scavenge them^52^. Elevated H_2_O_2_ levels can damage cellular components, including proteins, lipids, and DNA, impair growth, reduce root development, and decrease plant performance. The increase in H_2_O_2_ by SRB might trigger a defense response in the plant. This response can help the plant cope with oxidative stress and protect against potential pathogens or stressors. The effects of H_2_O_2_ on root growth and plant performance can be context-dependent, influenced by the specific species and concentration of SRB, as well as the overall plant health and environmental conditions. Different plant species may have varying sensitivities to H_2_O_2_, leading to diverse responses in root growth^53,54^. However, it is essential to mention that, in our study, H_2_O_2_ levels increased significantly by SRB. At the same time, it is true that an increase in H_2_O_2_ levels can be associated with stress responses and defense mechanisms in plants, it does not automatically classify SRB as pathogens. The increase in H_2_O_2_ induced by SRB could be part of a signaling cascade or a defense response triggered by the interaction between the bacteria and the plant. Although reduced levels in contaminated conditions could be attributed to this scenario, SRB may play a role in sequestering Cd, which can adversely affect the biology of SRB, leading to diminished recognition by the plant as a pathogen. As a result, the overall production of H_2_O_2_ is reduced under contaminated conditions (Fig. 7). In the absence of Cd, the introduction of sulfate-reducing bacteria (SRB) can induce mild oxidative stress in plants, leading to increased hydrogen peroxide (H₂O₂) production. This may occur as the plant’s immune response to SRB, recognized as a potential threat. SRB metabolism produces sulfide, which can further induce oxidative stress. Without Cd, the usual chelation and detoxification processes are not triggered, resulting in lower antioxidant defense and higher H₂O₂ levels. Additionally, the plant-bacteria interaction may activate H₂O₂-related signaling pathways, while the lack of Cd stress reduces the activity of antioxidant enzymes, contributing to H₂O₂ accumulation.

Catalase, as a crucial antioxidant enzyme, is involved in the detoxification of H_2_O_2_. This result is consistent with SRB’s low CAT activity. A decrease in CAT activity could result in reduced efficiency of H_2_O_2_ breakdown, leading to higher H_2_O_2_accumulation^55^. This could potentially contribute to oxidative stress and negatively impact root growth, as we observed in the current study. Conversely, the increased CAT activity induced by SP could enhance the breakdown of H_2_O_2_ and help mitigate oxidative stress. This suggests that SP treatment might improve the plant’s antioxidant defense system, allowing it to effectively manage and regulate H_2_O_2_ levels. However, the combined effect of SRB and SP on H_2_O_2_ levels could be influenced by the balance between H_2_O_2_ production and its breakdown by CAT enzymes. If SRB-induced H_2_O_2_ production outweighs the capacity of the plant’s CAT enzymes to break it down, H_2_O_2_ levels may accumulate, leading to oxidative stress and reduced root growth. On the other hand, SP treatment may enhance CAT activity, promoting efficient breakdown of H_2_O_2_and maintaining a balanced redox state^51^. SOD is an enzyme responsible for the breakdown of superoxide radicals (O^2−^) into H_2_O_2_ and oxygen. Low SOD activity in the presence of high H_2_O_2_ levels suggests that the plant’s antioxidant defense system may be compromised. Insufficient SOD activity could result in the accumulation of superoxide radicals, leading to increased conversion to H_2_O_2_and oxidative stress^56^.

### Mitigating the negative effects of cd stress on photosynthesis: role of SP and SRB combination in cd sequestration, light absorption, and photosynthetic efficiency

Cd stress can disrupt various physiological and biochemical processes, including photosynthesis. It can inhibit electron transport, damage chloroplast structures, and induce oxidative stress, reducing photosynthetic efficiency. While combined treatment of SP and SRB may positively affect plants under normal conditions, it appears that in the presence of Cd stress, the inoculation of SP and SRB calms the negative impacts on photosynthesis. Combined treatment may involve mechanisms that allow it to absorb or chelate heavy metals such as Cd. This can occur by binding Cd to specific compounds or functional groups in SP and sedimentation by SRB^57,58^. As a result, the available Cd may become sequestered or immobilized, reducing their availability for plant uptake. By absorbing Cd, SP can potentially prevent or reduce its toxicity in plants^59^. Cd contamination often results from accumulating Cd ions in plant tissues, leading to impaired physiological processes and cellular damage^60,61^. If SP treatment effectively limits the availability of Cd, it can help protect plants from the negative impacts of Cd stress. When the total absorption of light energy by PSII (Pi-abs) increases, it indicates that more light energy is being captured by the photosynthetic apparatus^62–66^. This increased light absorption can potentially lead to a higher availability of excitation energy for photochemistry, which may positively impact the overall efficiency of PSII, as reflected by F_v_/F_m_. Our results showed the same pattern, and SP in combination with SRB could increase both values (Fig. 6). However, in current research, SRB negatively affected F_v_/F_m_and Pi-abs values. It can be speculated that during a pathogen attack (here, hypothetical pathogen), the plant prioritizes the biosynthesis of defense-related compounds. At the same time, other cellular activities, such as those related to growth, are reduced. This leads to a decrease in photosynthetic rates until the growth of the pathogen is halted. The Pi-abs value serves as an indicator of a plant’s photosynthetic capacity and overall health. A higher Pi-abs value indicates improved photosynthetic performance and efficiency, while a lower value suggests potential limitations or stress on the photosynthetic apparatus. Therefore, according to these data, plants consider SRB a pathogen, and it is advantageous to co-apply with SP, which has shown positive effects. SP inoculation may contribute to an increased ABS/RC and ET0/RC by improving the ability of PSII to capture photons and absorb light energy. This could be due to various factors, such as the presence of specific compounds or functional groups in SP and SRB combination that enhance light absorption or modify the structural and functional properties of the photosynthetic apparatus^20,67,68^. The combination of SP and SRB treatments may have synergistic effects on the light absorption efficiency of PSII. SP treatment has previously been shown to improve light absorption efficiency, and SRB may further enhance this effect^41^. The presence of SRB might promote plant growth and metabolism, leading to increased chlorophyll content and improved light harvesting capacity. This combination might also have positive interactions that benefit PSII. For instance, SRB can promote plant growth by enhancing nutrient availability, particularly sulfur, essential for chlorophyll synthesis and photosynthesis. Improved plant growth and nutrient status can enhance photosynthetic performance and light absorption efficiency. In our study the enhancement of photosynthetic efficiency is in complete accordance with the reduction in Cd content in the SP and SRB co-treatment, which can reduce Cd accumulation in leaves and exert a positive effect on photosynthesis compared to other treatments (Fig. 6).

## Conclusion

Current experimental results demonstrate that, under non-contaminated conditions, SRB alone impedes plant growth (Fig. 6) and the antioxidative capabilities of plants. However, plants exhibited a response to stress signals, such as the generation of H_2_O_2_, suggesting the perception of SRB as a pathogen. At the same time, the introduction of SRB in Cd-contaminated soil may have reduced free Cd levels, perhaps due to its ability to produce sulfide radicals, resulting in Cd sedimentation. the application of SP may not only facilitate Cd remediation from the soil but could also potentially induce modifications in SRB-mediated mechanisms in plants, possibly leading to a reduction in their hypothetical pathogenic properties. This effect may be attributed to the upregulation of antioxidant enzymes in plants or the modulation of plant processes, including hormone signaling and (may) the production of metabolites with potential inhibitory effects on pathogens. It is also plausible that the influence of SP on the plant-SRB interaction holds significance, rather than the bacterium itself, as the simultaneous utilization of SP and SRB reduced Cd accumulation in lettuce leaves. Since SRB may contribute to the reduction of Cd absorption, its functionality in the soil environment and the potential for direct interference between SP and SRB could be minimized, although this remains a hypothetical scenario. Nevertheless, it is evident that the concurrent application of SRB and SP yields positive outcomes on various growth parameters, including leaf area and photosynthetic performance, particularly in contaminated soil (Fig. 7). Furthermore, the substantial decrease in Cd content observed in plants treated with both SP and SRB highlights the potential of this combined approach in promoting healthy plant growth in contaminated soils. However, additional comprehensive studies and the development of suitable methodologies are warranted to enhance germination rates and optimize the utilization of such soils. Together, *Spirulina* and SRB improve nutrient exchange and modify the rhizosphere, fostering a balanced microbial ecosystem that supports plant growth and enhances tolerance to stress conditions more effectively than either microorganism alone.

## Methods

### *Spirulina* cyanobacteria cultivation

*Spirulina platensis*(strain ISB88) was obtained from the Research Institute of Applied Sciences in Shahid Beheshti University branch of ACECR, Tehran, Iran. Cultivation and propagation were carried out in Zarrouk medium^69^ with a light/dark cycle of 16 h of light and 8 h of darkness at 23 ± 2 °C. To achieve rapid propagation, liquid Zarrouk medium was used, and after 12–16 days, the culture’s optical density (OD) at 600 nm (OD_600_) reached 0.7–1.

### Sulfate-reducing bacteria cultivation

The cultivation of SRB was performed using *Desulfovibrio desulfuricans*species in Postgate medium B^70^ at the Industrial Microbiology Laboratory, Shahid Beheshti University. Approximately 1 g of sediment samples from marshes was added to 50 ml of specialized bacterial growth medium to isolate SRB bacteria. The bottles were sealed to create anaerobic conditions and incubated at 30 °C for one week. In this culture medium, SRB cells grew and turned the culture black. The optical density of the grown bacteria was measured, and the culture was adjusted to a desired concentration (OD_600_ = 1) by adding more growth medium or allowing further growth. The bacterial culture was centrifuged at 5000 rpm for 10 min, and subsequently, the bacterial sediment obtained was carefully dissolved in 10 ml of sterile distilled water. Finally, the culture density was adjusted to 0.8 by adding water.

### Preparation of plant growth medium

The growth medium was prepared using a soil mixture of 50% coir pith and 50% perlite. Previous research has shown successful results using this growth medium for lettuce plants under saline stress^33^. To ensure rapid and better seedling growth up to the plant, the seedlings were initially maintained in seed trays and then transferred to pots.

### Application of sulfate-reducing bacteria, Spirulina, and cadmium in the culture medium

One day before seeding, SRB, with an optical density of 0.8 in distilled water, and SP, with an optical density of 0.8 in distilled water, were added to the dry soil. For Cd treatment, dry soil was incubated with CdNO_3_solution for 48 h^71,72^. The final concentration of 150 mg of CdNO_3_ per kg of dry soil was considered. The factors of soil type (with SP, with SRB, with both SP and SRB, and control (without bacteria)) and Cd concentration (NH_4_NO_3_ as 0 and 150 mg of CdNO_3_ per Kg of dry soil) were considered. The experiment had four replications for the control treatment (without Cd (only NH_4_NO_3_)) and Cd stress conditions. An additional nitrogen source in the control group was provided to the Cd-treated plants by supplementing the Hoagland solution^73^ with ammonium (NH_4_^+^).

### Sterilization of lettuce seeds

7–8 seeds were sown in each cell of the seedling tray. The seeds were rinsed with running water and then sterilized with 70% ethanol (1–2 min) and 1% sodium hypochlorite (3 min). After each stage, the seeds were rinsed with autoclaved distilled water^74^.

### Seeding in germination trays

The soil corresponding to each treatment was placed in the wells of germination trays and then sown in the soil. The germination trays were kept in a growth chamber with an average temperature of 23 ± 2 °C and a light-dark cycle of 16 h of light and 8 h of darkness. Irrigation was performed every other day with the Hoagland solution. The soil moisture was measured daily using a moisture meter and adjusted based on 60% water level.

### Transplanting seedlings to pots

Seedlings at the 3–4 leaf stage were subsequently transplanted into pots of identical size and conditions. Pots were irrigated every other day with Hoagland solution. Additionally, once a week, 20 ml of the respective bacteria were added to the soil of each pot based on the soil treatment (with SP, with SRB, with both SP and SRB, and control (without bacteria)).

### Sampling

Sampling was carried out 8 weeks after sowing from mature plants in CdNO_3_ and NH_4_NO_3_ with a minimum of 4 pots from each treatment, resulting in a total of 32 pots.

### Root length and leaf area measurement

Plants were photographed to measure root height and leaf area, and the images were analyzed using ImageJ 1.44p software (National Institutes of Health; Bethesda, MD)^75^.

### Relative water content measurment

Leaf samples from all experimental treatments were collected to measure the relative water content. Subsequently, the fresh weight of the samples was promptly determined. All samples were placed in distilled water in darkness for 24 h. The saturated weight of the leaves was measured, and the leaves were dried in an oven at 70 °C for 24 h. The dry weight of the leaves was then measured. The relative water content (RWC) in leaf samples was calculated using the following formula^76^:

RWC = (Fw - Dw / Sw - Dw) × 100.

Fw: Fresh weight of the leaves immediately after sampling.

Dw: Dry weight of the leaves after drying.

Sw: Saturated weight of the leaves after being placed in distilled water.

### Measurement of H_2_O_2_ concentration

For the measurement of H_2_O_2_concentration, the method described by^77^ was utilized. Fresh plant tissue weighing 500 mg was homogenized on ice with 5 ml of 1% (w/v) trichloroacetic acid. Subsequently, 500 µl of the supernatant was mixed with an equal volume (500 µl) of 10 mM potassium phosphate buffer at pH 7, followed by 1 ml of 1 M potassium iodide solution. The absorbance of the solution at a wavelength of 390 nm was measured using a spectrophotometer.

Standard solutions in the 0–100 µM range were prepared, and the corresponding standard curve was plotted based on the solutions’ absorbance at a wavelength of 390 nm. The H_2_O_2_ concentration of each sample was calculated using the formula derived from the standard curve. The concentration was then divided by the weight of the plant tissue to obtain the H_2_O_2_ content per gram of tissue.

### Measurement of catalase (CAT) enzyme activity

The CAT was extracted according to the method described by^78^. 0.25 g of plant tissue was finely powdered in liquid nitrogen. The powdered tissue was homogenized in 1.5 ml of cold 100 mM phosphate buffer (pH 7) containing 1% polyvinylpyrrolidone (PVP) and 1 mM EDTA. The homogenate was centrifuged at 10,000 g for 15 min at 4 °C. The supernatant was used for enzyme measurement.

The CAT activity was measured using the method described by^79^. 100 µL of the diluted enzyme extract and 1 ml of 50 mM potassium phosphate buffer (pH 7) were mixed, and the reaction was initiated by adding 100 µL of 100 mM H_2_O_2_. The changes in absorbance at 240 nm were measured for 2 min at 15-second intervals using a spectrophotometer.

The CAT activity (U.ml-1) was calculated using the following formula:

Enzyme activity (U.ml-1) = [Change in absorbance (Δ A) × Reaction mixture volume (1.2 ml) × H_2_O_2_ coefficient (2)] / [Extinction coefficient (39.4) × Path length (1 cm) × Extract volume (0.1 ml) × Δt (2)]

The specific activity of the CAT enzyme (U/mg protein) was calculated by dividing the enzyme activity by the total protein content.

### Measurement of superoxide dismutase (SOD) enzyme activity

The activity of SOD enzyme was determined using the method described by^80^. Fresh or frozen plant tissue weighing 0.5 g was homogenized with 3 ml of 50 mM sodium phosphate buffer at pH 7.8, containing 2% PVP and 1 mM EDTA. The homogenate was centrifuged at 13,000 g for 10 min at 4 °C. The reaction mixture buffer consisted of 50 mM potassium phosphate buffer at pH 7.8, 0.1 mM EDTA, 75 µM NBT (nitro blue tetrazolium), 13 mM methionine, and 4 µM riboflavin.

To initiate the reaction, 100 µl of the sample solution was added to 3 ml of the reaction mixture buffer, and the combination was exposed to a 40-watt fluorescent light for 8 min. After 8 min, the absorbance of the reaction mixtures at a wavelength of 560 nanometers was measured using a spectrophotometer. The control sample consisted of 3 ml of the reaction mixture without the extract, which was kept in the light.

The activity of SOD enzyme was calculated using the following formula:

SOD Activity (U/mg protein) = 2 × [(Absorbance Control - Absorbance Sample) / Absorbance Control] / Total Protein.

### Slow and fast induction of chlorophyll fluorescence

The maximum quantum efficiency of photosystem II (F_v_/F_m_), intact leaves were utilized, and an advanced chlorophyll fluorescence imaging system was employed for precise measurements (Handy Fluor-Cam FC 1000-H; Photon System Instruments, Brno, Czech Republic). The measurements were conducted 8 weeks after sowing, following 20 min of dark adaptation. Two series of fluorescence data were collected: one during short flashes in darkness (representing minimum fluorescence, F0), and the other during a saturating light flash (F_m_) with a light intensity of 3900 µmol m^−2^ s^−1^. Variable fluorescence (F_v_) was calculated as the difference between F_m_ and F_0_. The average data and standard deviation for F_v/_F_m_were recorded using version 7 of the Fluor-Cam software^33^.

To analyze the polyphasic chlorophyll fluorescence (OJIP) transient, a convenient fluorometer system (FluorPen FP 100-MAX, Photon Systems Instruments, Drasov, Czech Republic) was utilized. Similar to the gradual induction of chlorophyll fluorescence, the measurements for the OJIP transient were performed on plants that underwent a 20 min period of dark adaptation. A saturating light with an intensity of 3000 µmol m^−2^ s^−1^ induced the transient. The fluorescence data were collected at various time points, including fluorescence intensities at 50 µs (F0), 2 ms (FJ or J-step), 60 ms (FI or I-step), and the maximum fluorescence (F_m_).

To assess the performance index on an absorption basis (Pi-abs), the following equation was employed:

Pi-abs = [1/ (ABS/RC)] × [*F*_*v*_*/F*_*m*_ / (1-*F*_*v*_*/F*_*m*_)] × [*ψ*^*0*^*/ (1-ψ*^*0*^)]

Here, ABS/RC represents the energy fluxes per reaction center (RC) for energy absorption, and ψo denotes the probability (at t = 0) of electron transport beyond Quinone A^−^. Additionally, other calculated data such as specific energy fluxes per reaction center (RC) for energy absorption (ABS/RC), trapped energy flux (TR0/RC), electron transport flux (ET0/RC), and dissipated energy flux (DI0/RC) were derived based on the parameters obtained from the OJIP fluorescence transient^33^.

### Statistical analysis of data

The data obtained from the experiment were analyzed using analysis of variance (2-way ANOVA) based on a completely randomized design (CRD) with a minimum of 4 replicates per treatment. The effects of the treatments on the measured traits were evaluated using statistical software such as SPSS^81^and PRISM^82^. The means of the treatments were compared using the Tukey method, and the treatments were grouped using the 2-way ANOVA at a significance level of *P* ≤ 0.05. This level was considered as the threshold for determining the significance of differences.

## Data Availability

The datasets generated and/or analyzed during the current study are available from the corresponding author on reasonable request.
